# Deep Learning in Medical Hyperspectral Images: A Review

**DOI:** 10.3390/s22249790

**Published:** 2022-12-13

**Authors:** Rong Cui, He Yu, Tingfa Xu, Xiaoxue Xing, Xiaorui Cao, Kang Yan, Jiexi Chen

**Affiliations:** 1 College of Electronic and Information Engineering, Changchun University, Changchun 130022, China; 2 Jilin Provincial Key Laboratory of Human Health Status Identification and Function Enhancement, Changchun University, Changchun 130022, China; 3 Image Engineering & Video Technology Lab, School of Optics and Photonics, Beijing Institute of Technology, Beijing 100081, China; 4 Beijing Institute of Technology Chongqing Innovation Center, Chongqing 401120, China

**Keywords:** medical hyperspectral imaging systems, disease diagnosis, deep learning

## Abstract

With the continuous progress of development, deep learning has made good progress in the analysis and recognition of images, which has also triggered some researchers to explore the area of combining deep learning with hyperspectral medical images and achieve some progress. This paper introduces the principles and techniques of hyperspectral imaging systems, summarizes the common medical hyperspectral imaging systems, and summarizes the progress of some emerging spectral imaging systems through analyzing the literature. In particular, this article introduces the more frequently used medical hyperspectral images and the pre-processing techniques of the spectra, and in other sections, it discusses the main developments of medical hyperspectral combined with deep learning for disease diagnosis. On the basis of the previous review, tne limited factors in the study on the application of deep learning to hyperspectral medical images are outlined, promising research directions are summarized, and the future research prospects are provided for subsequent scholars.

## 1. Introduction

Medical imaging refers to an imaging technology mainly used to assist in clinical work, often in the initial detection, treatment, and diagnosis of many diseases and the guidance of operations. Modern medical imaging mainly uses magnetic resonance imaging (MRI), X-ray, optical coherence tomography (OCT), ultrasound, or a combination of several techniques. These modern medical imaging modalities have had a profound impact on the diagnosis of diseases and have led to the development of more imaging techniques for clinical examinations. Deep learning has made significant progress in other medical image processing, such as optical coherence tomography (OCT), a non-invasive imaging technique that scans the subject to obtain three-dimensional high-resolution images, mainly for fundus retinal imaging, etc. The main algorithms currently focus on convolutional neural networks, support vector machines (SVM), etc. However, most of these imaging techniques are expensive and can even be harmful to the human body. Therefore, it is important to obtain an inexpensive and non-invasive imaging technique for medical images.

Hyperspectral imaging (HSI) originally originated from remote sensing and was used by NASA for various applications with richer spectral information as well as spatial information than conventional optical images. It has been broadly applied in diverse fields of remote sensing data [1,2], agriculture [3,4], image enhancement [5], horticultural protection [6,7], disaster monitoring [8], food safety and assessment [9,10], and medicine [11,12,13], showing its great potential.

The hyperspectral images consist of aligning various images in a narrow band of adjacent wavelengths or spectra and reconstructing the reflection spectra of all pixels in that band to obtain three-dimensional hypercube data. The obtained spatially resolved spectra give access to diagnostic information about tissue physiology, morphology, and composition; thus, enabling the non-invasive observation of biopsies, histopathological and fluorometric analysis, and increased understanding of the biology of the disease. Hyperspectral imaging is one of the developing imaging techniques in imaging modalities, and various spectral imaging systems have been investigated over the past decades to be used in the assessment of various biological organs and tissues. From the predominant and traditionally used whiskbroom, push broom, staring, and snapshot imaging systems developed to the fluorescent hyperspectral imaging systems, multispectral analysis, as well as separation techniques, have been implemented. The advantages of handheld hyperspectral imagers that use single-image fast spectral capture and are capable of rapid imaging are also gradually being applied in research. Spectral imaging techniques in biomedicine have attracted more attention and have gained an important position in research.

The application of medical hyperspectral imaging (MHSI) for the diagnosis of various diseases has given rise to a variety of algorithms that combine with it to enable more accurate and efficient diagnosis and classification detection of various diseases. Machine learning (ML) typically employs data and statistical models that learn and recognize patterns to accomplish particular tasks. In medical hyperspectral image (MHSI) processing, ML is mostly used in combination with MHSI for disease diagnosis and classification, detection, and segmentation of pathological images, including K-Nearest Neighbor [14] (KNN), Linear Discriminant Analysis [15] (LDA), and Support Vector Machine (SVM) methods. However, the MHSI application of deep learning (DL) methods has been increasingly proposed [16] and studied by academics. It has produced positive results ever since the large-scale image classification challenge in 2012, when a network of CNNs was introduced on the ImageNet dataset and made significant progress. For example, a study in 2017 [17] used a convolutional neural network (CNN) to classify blood cells in MHSI, distinguishing red cells as well as white cells. In medical hyperspectral datasets, CNNs clearly outperform the conventional SVM in classification accuracy, demonstrating the huge promise of deep learning (DL) in this field [18].

Over the past decade, a number of pioneers in this field have assembled correlative references and compared the parameters of common medical-imaging techniques [19]. These studies illustrate the convenience that hyperspectral imaging brings to the field of medical bioengineering in comparison to traditional optical imaging methods, allowing for a greater wealth of information than was previously available. One study discussed the ongoing advancement of bio-medical hyperspectral systems and the parallel of the approaches to imaging and presented the current challenges [13]. This study presents a contributes to the extant literature by providing a well-balanced integration of academic opinion, and practical perspectives. Subsequent articles have combined techniques such as acquisition mode, spectral range, and spatial resolution, and measurement mode to classify MHSI. Methods for image analysis, as well as disease diagnosis and surgical guidance, were also summarized [11]. Along with the growth of deep learning, it has attracted a lot of followers to study this type of field. Some authors have also made a summary of medical hyperspectral imaging in the field of deep learning and discussed the deep learning approach and how this approach is applied in the medical field [20].

However, the existing studies of hyperspectral medicine are fragmented and not comprehensive, while DL is rapidly emerging, and the related studies are complicated but lack a theoretical foundation. Therefore, a clear context is needed to link hyperspectral images, hyperspectral medicine, and DL. An overview of the development experience of other associated and also relatively mature research fields will provide a reference for later scholars to develop this nascent field. Through a review of relevant literature readings, a synthesis of key technical insights from current research, and a revelation of major research trends in this field, this study intends to answer the following research areas: the development of hyperspectral imaging systems; the mainstream architecture of deep learning for MHSI applications; and the problems addressed in medical diagnosis.

With the development of DL in MHSI, more attention will be drawn to its application. It will have a remarkable influence on the medical field, especially on the diagnosis of diseases and the guidance of surgery. This paper introduces various imaging systems for hyperspectral imaging as well as the usage of deep learning to classify, segment, and detect medical images and also gives a brief introduction to the application of hyperspectral imaging in medical applications.

## 2. Hyperspectral Imaging Technology

### 2.1. Imaging Principles and Techniques

**Figure 1:**
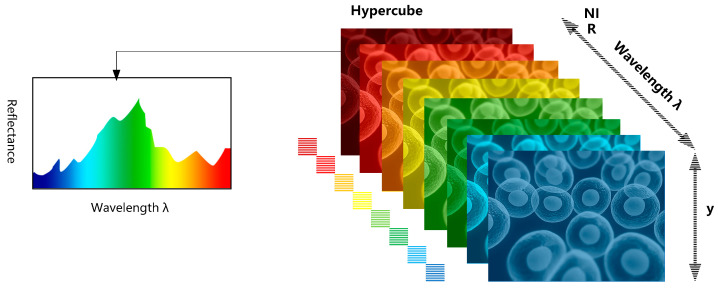
Spectral data cube.

Hyperspectral imaging is a modality that combines imaging with spectroscopy. It usually covers a continuous part of the spectrum and provides continuous scanning imaging of tens or hundreds of spectral ranges at ultraviolet (UV), visible (VIS), infrared, and even mid-infrared wavelengths [12]. As illustrated in Figure 1, which contains both two-dimensional spatial and one-dimensional spectral information, or as a superposition of several two-dimensional images [21]. It is possible to obtain the reflectance, absorption, or fluorescence spectra of every pixel in the image by this technique. It has a richer spectral band as well as a higher spectral resolution than conventional RGB images and grayscale maps. It can see changes in objects that are not visible with conventional imaging techniques and captures minor spectral nuances in response to different pathological conditions.

**Figure 2:**
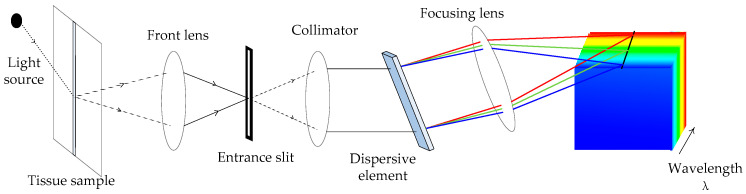
Schematic diagram of push-scan hyperspectral imaging system.

The system mechanism of HSI is elucidated by the typical push broom hyperspectral system principle [11], as shown in Figure 2. First, a light source is irradiated to the spatial information and passes through the front lens into the slit, where different wavelengths of light are bent to varying degrees. Then, each pixel point in that dimension is shone on the detector through dispersion devices such as gratings and prisms to split the light into narrow spectral bands. Each row of sample space information is treated as a two-dimensional image and imaged on the detector array. Moving through the plane by a mechanical push sweep, the HSI camera collects adjoining two-dimensional images, resulting in a hypercube with two spatial dimensions and one spectral dimension.

### 2.2. Imaging System

A typical medical hyperspectral imaging system can be categorized into: an optical acquisition instrument; spectral spectrometer; detector; system control; and data acquisition module [13]. Optical acquisition instruments indicate devices that produce images on a spectral spectrometer, such as a camera-like instrument that has a real image or a microscope that has a virtual image. After collecting and summarizing, the HSI imaging systems for medicine are listed in Table 1.

#### 2.2.1. Acquisition Mode

**Figure 3:**
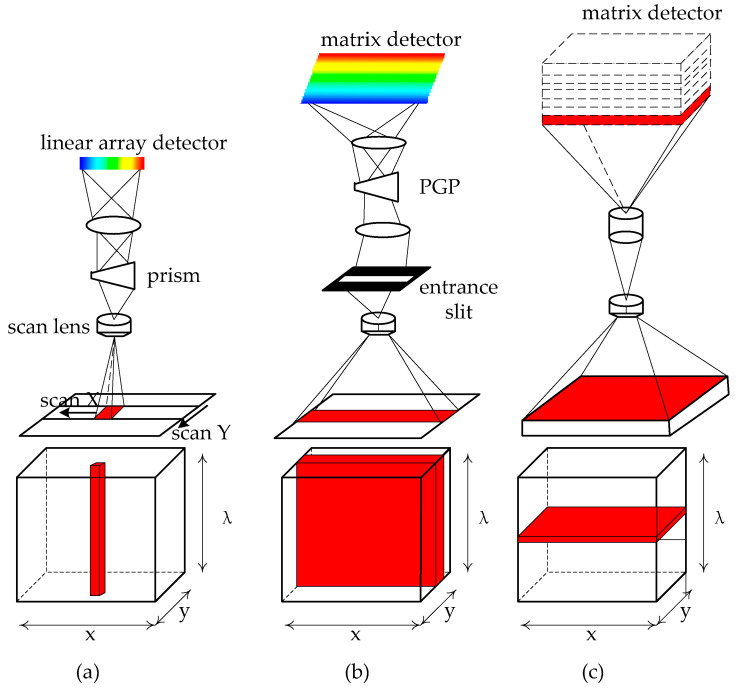
Typical spectral imaging methods. (**a**) Whiskbroom. (**b**) Push broom. (**c**) Staring.

According to the acquisition methods [22] of hyperspectral systems for spectral as well as spatial information, hyperspectral systems are classified into four typical methods: whiskbroom; push broom; staring; and snapshot [13], as shown in Figure 3.

Whiskbroom imaging systems, also known as point-scan methods, typically employ a rotating scanning mechanism that sweeps a single point clockwise along the spatial dimensions (x and y) and spectral dimensions (λ). The signal to the biological tissue is sequentially passed through the swivel scanning mirror and the front optical system, and then the spectral spectrometer is spectroscopic and imaged on the CCD. The spectral image data cube (x,y,λ) can be obtained by the spatial dimension (x and y) along with the spectral dimension (λ) obtained from the two-dimensional scene.

1. Push broom

The push broom imaging system is also known as the line-scan method. Unlike the point scan, the line scan can simultaneously scan to obtain the spatial information of the slit and sweep once for the spectral info of every spatial point. The biological tissue light signal will pass through the objective lens, the incident slit, the collimation module, and then the dispersive element to complete the spectroscopy and image on the CCD in turn.

**Figure 4:**
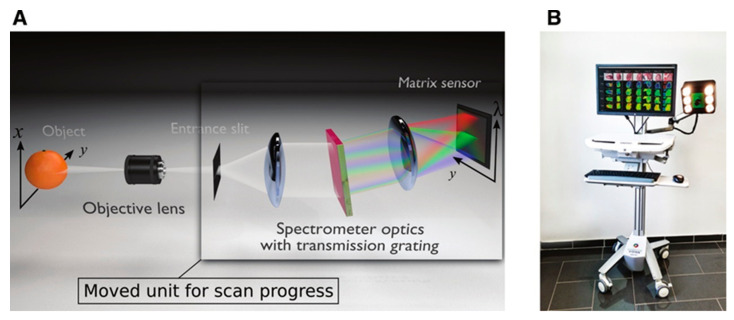
Push broom TIVITA tissue camera, (**A**) schematic diagram of push broom spectroscopy system; (**B**) hyperspectral camera mounted on a medical vehicle [23].

The CMOS push broom hyperspectral camera, TIVITA, is often used in medical biological tissue detection to generate HS images at a spectral resolution of 5 nm captured at a spectral range of 500–1000 nm spectral range, generating a 640 × 480 × 100 data cube with an acquisition time of about 6–7 s. It can also be used for RGB image reconstruction based on HSI. The diameter between the camera and the tissue depends on the objective lens, which is usually between 30–50 cm. For acquisition, the operating room lights are turned off to avoid interference from external light sources and installed on a mobile and agile medical system, as shown in Figure 4B and described by the following references in detail [23,24].

Push broom imaging usually acquires a greater amount of light than whiskbroom imaging, providing a longer exposure time and higher spectral resolution for the detector [25]. These two imaging systems do not display the spectral image in real time, which is derived from spectral calculations when the scanning of the corresponding points and regions is completed;

2. Staring

The staring-type imaging system, also called the spectral scanning method, can simultaneously capture a single-band two-dimensional grayscale image of complete spatial information with spectral data cubes obtained by sweeping the spectral domain in multiple bands. The staring type uses filters such as liquid crystal tunable filters (LCTF) and acousto-optic tunable filters (AOTF) [26] to complete scanning of the spectrum, followed by a focusing optical system that is filtered to produce a narrow spectral band and imaged in the detector focal plane. Therefore, two-dimensional image information in one band is usually captured, and the information imaged in different bands is stacked to form an image cube [27]. The cube constructed by wavelength scanning offers the merit of revealing spectral information in real-time, which is important for targeting and focalization [28]. Due to the short acquisition time, it is easy to couple with some optical instruments, such as cameras, endoscopes, or microscopes, and is widely used in biomedicine for detecting ex vivo tissues, etc.;

3. Snapshot

Snapshot imaging systems can record spatial as well as spectral information on the detector in a single exposure area, and the snapshot mode does not require scanning in spatial and spectral dimensions, resulting in limited spatial as well as spectral resolution. Consequently, for a given CCD, spectral sampling can be compensated for by increasing the sampling space [29]. The snapshot imaging system differs from the whiskbroom, push broom, and staring modes in that the imaging regime does not require scanning to be imaged and can engage both remapped and scattered images to be imaged onto the CCD detector [30]. Thus, the obtained data, through direct and simple processing, can construct a spectral data cube. Nevertheless, the strength of this image is that it allows for rapid experiments and is usually suitable for rapid process studies, such as endoscopic inspection.

**Table 1:** Application areas of hyperspectral imaging systems and medicine.

Reference	Spectral Range (nm)	Spectral Resolution/nm	Detector	Spectral Spectroscopy	Acquisition Mode	Applications
[31]	450~900		CRI Maestro imaging system	LCTF		Tumor margin classification
[32]	430~680		Monochromatic CCD-camera			In vivo tumors
[33]	450~900	5	CRI Maestro imaging system	LCTF		Head and neck cancer
[34]	500~995	5	TIVITA Tissue Camera		Push broom	Ex vivo kidneys classification
[35]	350~1000	>1	Micro-hyperspectral imaging system	PGP		Stomach Cancer Classification
[17]			Silicon charge-coupled devices	LCTFs		Blood cell classification
[36]	400~720		CCD	LCTF		Blood cell classification
[37]	500~1000	5	TIVITA Tissue Camera		Push broom	Tissue classification
[38]	400~1000	2~3	VNIR camera, HELICoiD demonstrator, Si CCD	LCTFs	Push broom	Brain cancer detection
[39]	430~920		Hyperspectral line-scan camera (IMEC)		Push broom	Colon cancer classification
[40]	477~891		SICSURFIS Spectral Imager	FPI	Hand-held	Skin Tumors
[41]	450~950	8	Snapshot HS camera		Snapshot	Skin Cancer
[42]	400~1000	2.8	CCD		Push broom	Breast cancer cell detection
[43]	450~950		CRI Maestro imaging system	LCTF		Head and neck cancer
[44]	500~1000	5	TIVITA Tissue Camera		Push broom	Esophageal cancer classification
[45]			Spatial-scanning hyperspectral endoscope (HySE)		Push broom	Esophageal cancer
[46]	450~950		CCD	FPI	Snapshot	Skin feature detection
[47]	400~1000	2.8	Microscopic HS camera, CCD	PGP	Staring	Brain cancer classification
[48,49]	450~900	5	CRI Maestro imaging system	LCTF		Head and neck cancer
[50]	500~1000	5	TIVITA Tissue Camera		Push broom	Surgical Instruction
[51]	400~1000	2.8	CCD		Push broom	Brain tissue
[52]	486~700		SnapScan hyperspectral camera			Head and neck cancer
[53]	450~900		CRI Maestro imaging system, CCD	LCTF		Head and neck cancer
[54]	400~1000 900~1700		Hyperspectral cameras		Push broom	Tongue tumor detection
[55]	550~1000	7.5	CCD	AOTF		Melanoma segmentation
[56]	500~1000	5	TIVITA Tissue Camera		Push broom	
[57]	500~1000	5	TIVITA Tissue Camera		Push broom	Tissue segmentation
[58]	450~680		CMOS	LCTF		Stomach Cancer Classification
[59]	900~1700		InGaAs Hyperspec^®^		Push broom	Stomach Cancer Classification
[60]	450~950		CRI Maestro imaging system, CCD	LCTF		Head and neck cancer
[61]	510~900	6~10	Compact imaging system	FPI	Hand-held	Diabetic skin complications
[62]	500~1000	5	HSI Laparoscope	Monochromator	Push broom	Excised tissue reflectance measurement

Note: LCTF, liquid crystal tunable filter; PGP, prism-grating-prism; AOTF, acoustic-optical tunable filter; FPI, Fabry–Pérot interferometer.

#### 2.2.2. Fluorescence Hyperspectral Imaging System

The CRI Maestro (Caliper Life Sciences, Inc. (Nasdaq: CALP), United States of America) Hyperspectral Imaging System [53,63,64] allows the acquisition of hyperspectral images of in vitro surgical specimens. Spectral scans were usually performed using a liquid crystal tunable filter (LCTF) and a 300-W photocatalytic xenon light source (Cermax-type, 300-Watt, Xenon light source, Excelitas Technologies Corp, America) [65]. The system combines multispectral imaging and its analysis to acquire each pixel point in the spectrum range from visible through near-infrared. Combined with the spectral information from the object, it achieves multispectral analysis, separation, and other techniques to become an in vivo fluorescence imaging technique with high accuracy and sensitivity.

#### 2.2.3. Handheld Hyperspectral Imaging System

Unlike traditional push broom hyperspectral imagers, handheld hyperspectral imagers use fast spectral capture of a single image and are capable of rapid imaging. The small form factor and simple operation reduce the complexity of handling common imaging.

**Figure 5:**
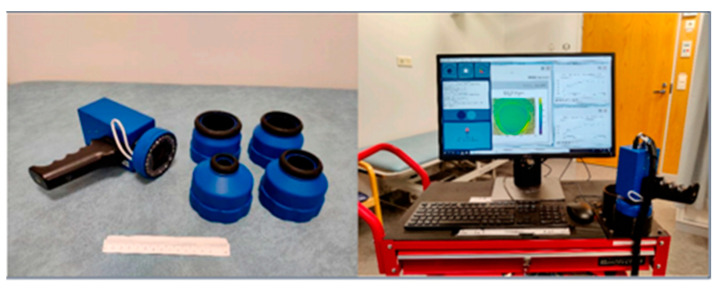
SICSURFIS handheld hyperspectral imaging system [40]. The article [66] describes the principle and testing of the first phase of a three-stage pilot of this imaging system, focused on the intricate skin surface. The device is still a prototype and ongoing refinement is needed before it can be used for clinical applications.

Raita-Hakola et al. [66] presented the SICSURFIS handheld hyperspectral imaging system [40], as shown in Figure 5. It is a compact, handheld, piezoelectrically driven metallic mirror-based Fabry–Pérot interferometer (FPI) hyperspectral imager. It consists of a prototype handheld piezoelectric metal-mirror FPI hyperspectral imager, an RGB sensor, and an LED light source. The light source is a series of three purposely selected nine LEDs that can deliver light in the range of white to 940 nm. It is almost as fast as the snapshot spectral imager adapted to complex skin surfaces, and allows for stereoscopic imaging by tilting at given angles. The imager thus provides spectral images at different angles for photometric stereo calculations, allowing for skin surface modeling on each captured wavelength. As shown in Figure 5, left below, the mode, spectral separator, and LED of the spectral imager’s HSI are all independently controllable and can be configured arbitrarily and efficiently by software.

**Figure 6:**
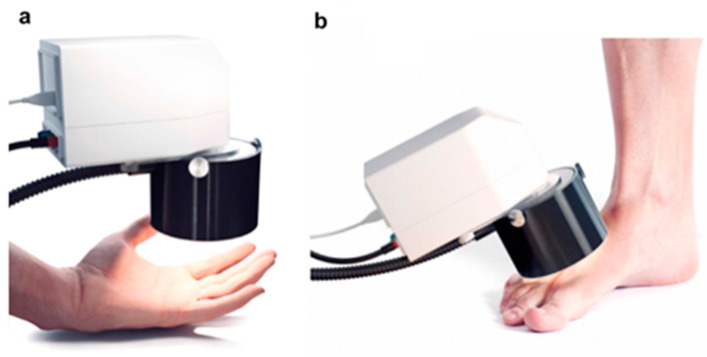
Compact handheld hyperspectral imaging system [61]. The proposed imaging system [67] was designed to enable quantitative diagnosis and visualization of human skin. This includes 2-dimensional mapping of skin chromophores, mapping of hemo–oxygen dynamics, and assessment of skin perfusion.

Besides the SICSURFIS handheld hyperspectral imaging system described above, another one is the compact imaging system [61,67]. This imaging system is built on a hyperspectral snapshot camera and uses FPI to provide a spectral resolution of 6–10 nm in a wavelength spectrum of 500–900 nm. As shown in Figure 6, this system can image randomly selected skin areas, where (a) is the detection of the skin of the palm of the hand and (b) is the detection of the dorsum of the foot in diabetic patients.

## 3. Medical Hyperspectral Image Analysis

Analysis of acquired hyperspectral images, especially medical hyperspectral images, can extract important information about diagnosis and treatment from some tissues and cells and is therefore very important for medical diagnosis and clinical applications. At the same time, since hyperspectral images usually also contain visible spectra and hundreds of spectral bands, they are regarded as a hypercube, which provides rich spectral information for image analysis and has the advantage of high spatial and high resolution to obtain more useful information, but at the same time, due to the high dimensionality, it is also more difficult to analyze, which can lead to data redundancy and dimensional disaster. Band selection can solve the problem of dimension disaster to a certain extent. Table 2 shows a comparison of the six band selection methods. Table 3 lists the image preprocessing operations used in the literature; Table 4 lists the deep learning architectures used in the literature.

### 3.1. Image Pre-Processing with Spectra 

#### 3.1.1. Normalization

After the acquisition of hyperspectral data, since the data have factors such as high-level dimensionality, intra-image band redundancies, and instrument noise, they need to be processed by some common preprocessing algorithms to obtain the hyperspectral data, which removes unnecessary noise.

The hyperspectral radiation observations are normalized to eliminate the spectral inhomogeneity and dark current effects of the illumination device. Spectral features based on uniform reflectance will be obtained for feature extraction. First, the raw radiation data are converted to normalized reflectance [68,69,70] using (1):

(1) $$I_{ref}=\frac{I_{raw}-I_{dark}}{I_{white}-I_{dark}}$$

where $I_{ref}$ is the acquired normalized reflectance; $I_{raw}$ is the original HS image; $I_{white}$ is the white reference image; and $I_{dark}$ is the dark reference image acquired using the acquisition system.

One of the most popular normalization techniques is Standard Normal Variate (SNV). SNV is usually applied to resolve scattering effects caused by the presence of particles of different sizes on the surface of an object, to reduce the inhomogeneity of the particles, and to resolve the effects caused by the NIR diffuse reflectance spectrum. SNV usually deals with one of the spectra, and its Equation (2) shows the transformed spectrum:

(2) $$x_{SNV}=\frac{x-\bar{x}}{\sqrt{\frac{\sum_{k=1}^{m}{\left(x_{k}-\bar{x}\right)}^{2}}{\left(m-1\right)}}}$$

where $\bar{x}=\frac{\sum_{k=1}^{m}x_{k}}{m}$; *m* is the number of wavelength points; *k* = 1, 2, ..., *m*.

#### 3.1.2. Smoothing Denoising

Spectral noise is present in the discovery of spectral features, and the HS sensor has a poor response across some bands, which should be removed. Smoothing filters are then used to filter the HS data [71] to diminish the random spectral noise in the remaining spectral bands. Smoothing filtering is the simplest and most effective method to eliminate noise, and algorithms such as window shifting and least squares are usually used, in which Savitzky–Golay [72] smoothing can greatly preserve data characteristics such as relative extremes and widths and accomplish smooth denoising of the original spectrum.

#### 3.1.3. Wave Selection

The choice of waveband [73] is an important tool and probably the most effective and direct method that can alleviate hyper-spectral data redundancy. It aims to pick a tiny subset from the hyperspectral bands, i.e., to select some information-rich and distinctive features from the original hyperspectral image cube, which reduces the calculated cost and maintains the physical characteristics of the bands [74].

The selection of hyperspectral bands in the current study is broadly divided into six types: ranking-based; search-based; clustering-based; sparsity-based; embedded learning-based; and hybrid scheme-based [74]. Since clustering algorithms only consider the redundant information of spectral bands and ignore the amount of information in the subset of bands, Wang et al. [75] developed a new method to select bands by an adaptive subspace partitioning strategy and achieved good results in terms of accuracy as well as efficiency. Sun et al. [76] proposed rapid and potential spectral bands for low-rank subspace clustering selection, with higher classification accuracy and lower computational cost as the end result.

**Table 2:** Comparison of six band selection methods.

	Principle	Advantages	Disadvantages	Differences and Similarities
Ranking-based	Use a suitable function to quantify the amount of information in each band, and then select the top subset of bands according to their importance	Low computational complexity and fast execution of calculations for larger hyperspectral datasets	Correlation between bands is often not considered	Search-based, sparsity-based, and embedding-learning band selection methods are all optimization problems with objective functions; ranking-based and clustering-based band selection methods are all based on the importance of bands. And all band selection methods are designed to select the combination of bands with high information content, low correlation between bands, and best class separability.
Search-based	The optimization problem of the criterion function is a multi-objective optimization to find the optimal frequency band	Only individual bands are considered, ignoring the entire subset of bands optimized	Computationally intensive and difficult to apply in practice	
Clustering-based	The representative subset of frequency bands in the cluster of the component group	Entire subset of bands can be optimized; less affected by noise; simple algorithm	Poor robustness, easy to fall into local optimal solutions	
Sparsity-based	Obtaining representative bands by dealing with sparsely constrained optimization problems	Can reduce the complexity of hyperspectral data processing; reduce storage space; improve model interpretability	Difficulty in automating model applications; uncertainty in model processing performance	
Embedded learning-based	Optimize the objective function of a specific model and select the appropriate spectral band	Avoids repetitive training of the learner for each subset of bands	Performance-dependent parameter tuning and difficult objective function construction	
Hybrid scheme-based	A synthesis of several band selection algorithms	Can find the best combination of frequency bands to get the least number of useful bands	Algorithm complexity	

#### 3.1.4. Feature Dimensionality Reduction

Feature downscaling, in other words, feature extraction, is also an important tool. However, feature extraction is the transformation of the primitive hyperspectral data by a linear or nonlinear mapping into lower dimensions, and the effective information is retained for subsequent analysis. Examples of typical methods involve principal component analysis (PCA) [77,78], linear discriminant analysis (LDA) [79], minimum noise fraction (MNF), and independent component analysis (ICA).

The principal component analysis has been the best-used method to decrease the dimensionality of hyperspectral features, improving interpretability without losing much information. It is a statistical technique that retains the maximum amount of information and eliminates redundant noise and data. The MNF transform is intrinsically two simultaneously cascaded PCA transforms designed to decrease spectrum dimensionality and separate noise out of image data. ICA enables spectral features that are as separate as possible, which is also a useful extension of principal component analysis. The critical idea in the independent component analysis is to assume that the data are amalgamated linearly across a set of individual sources and decompose them in terms of the statistical independence of the cross-information measures.

**Table 3:** Methods of image pre-processing.

	Normalization	Smoothing Denoising	Wave Selection	Feature Dimensionality Reduction	Calibration	Remarks
[43]	Normalized reflectance spectra					
[31]	Normalized reflectance spectra					
[33]	Normalized reflectance spectra					
[63]	Normalized reflectance spectra					Glare Removal
[34]	Normalized reflectance spectra	Savitzky–Golay smoothing				Manual background segmentation, automatic region of interest (ROI) selection
[80]				PCA		
[35]		Savitzky–Golay smoothing		PCA		First-order derivation for spectral dimension preprocessing
[17]				PCA		
[36]				PCA		
[37]	SNV					
[81]	SNV					
[82]	Normalized reflectance spectra			PCA		
[38]	Normalized reflectance spectra			Fixed Reference t-Distributed Stochastic Neighbors Embedding		HySIME noise filtering and extreme noise band Removal and spectral averaging
[83]	Normalized reflectance spectra			PCA		
[39]	Normalized reflectance spectra			PCA		
[84]					Shannon entropy	
[40]						Machine learning pre-processing
[85]	Normalized reflectance spectra			PCA		Singular Spectrum Analysis (SSA)
[41]	Normalized reflectance spectra	Smoothing filter noise processing				
[86]				PCA		
[31]	Normalized reflectance spectra					
[42]	Normalized reflectance spectra					
[87]	Normalized reflectance spectra	Smoothing filter noise processing				
[88]				ICA		K-means
[43]	Normalized reflectance spectra					
[44]	Normalized reflectance spectra					
[45]	Normalized reflectance spectra					
[89]	Normalized reflectance spectra					
[90]	Normalized reflectance spectra		ACO			Band Selection for Ant Colony Optimization (ACO)
[91]				PCA		
[46]	Normalized reflectance spectra			PCA		
[47]					Ratio between original Image and reference image	
[48]	Normalized reflectance spectra					
[51]	Normalized reflectance spectra			PCA		
[52]				PCA		
[49]	Normalized reflectance spectra	Smoothing filter noise processing				
[53]	Normalized reflectance spectra					
[54]				PCA		
[55]				PCA		
[56]		Median Filter				
[57]	Normalized reflectance spectra	Savitzky–Golay smoothing, gaussian filtered spatial smoothing		PCA		Outlier removal, background recognition
[92]	Standard normalization transformation	Gaussian filtered spatial smoothing				
[59]	Normalized reflectance spectra					
[60]	Normalized reflectance spectra	3-order median filter, curvature correction	GFP bands removal			Background removal
[61]	Normalized reflectance spectra					
[62]	Normalized reflectance spectra					

### 3.2. Classification

The categorization of medical highlight images (MHSI) represents an area where medical analysis was first applied, and now MHSI is becoming increasingly popular for medical diagnostic applications. Hyperspectral images with high resolution can provide richer spectral features for classification tasks, and this technique is mostly used for cancer detection and classification as well as for cell classification [93]. Previously traditional machine learning methods were often used for classification of medical hyperspectral images, ML uses data and statistical models for learning and recognition tasks and can make decisions with supervision or unsupervised. For example, Torti et al. [82] first used a supervised classification algorithm consisting of PCA, SVM, and KNN for classification and then used it in combination with a K-mean clustering algorithm (K-means) for the final weighted classification to correctly classify normal and cancerous tissues; Fabelo et al. [38] used a combination of supervised as well as unsupervised methods, using SVM for supervised pixel classification, then taking a t-Stochastic Neighbors Embedding dimensionality reduction algorithm, and finally the segmentation maps generated by combining unsupervised clustering were accurately identified at the margins of the neoplasms.

DL is a deep neural network-based approach. Compared with ML, DL does not need to set the features manually, and obtains great learning ability by increasing the number of layers of the network and calculating the weight parameters automatically, and then continuously learning the features of various data. As an end-to-end network model, it has advantages in image processing and is widely used in the classification of hyperspectral images. In traditional machine-learning algorithms for classification, the features for classification are represented by a one-dimensional vector. In contrast, HSI is multidimensional data consisting of two-dimensional image information and one-dimensional spectral information, and the amount of information contained in images at different wavelengths is different. Each image element is composed of hundreds of spectral bands, containing rich spectral features. So, it is necessary to downscale the multidimensional data during processing; then, the final extracted features will only contain spectral information, and spatial information will be ignored. Therefore, more and more scholars focus on the dual branch structure of simultaneous extraction of spatial as well as spectral features. Using deep learning methods, one can not only extract features in the spatial dimension by convolution but also have the convolution kernel slide into the spectral dimension to extract high-level spectral features. This method of simultaneous extraction of spatial information from hyperspectral images along with spectral features makes the extracted information richer and makes operations such as classification more accurate.

In contrast to the use of deep learning in other fields, the maturation of deep learning for MHSI has taken some time. Earlier, MHSI used artificial neural networks to classify cancer, and Nathan et al. [83] used an algorithm combining hyperspectral imaging with machine learning, i.e., using support vector machines (SVMs) and artificial neural networks (ANNs) to distinguish between different types of cancer.

Recently, convolutional neural networks (CNNs) have been largely used for the classification of MHSI. Huang et al. [36] 2018 proposed the extraction of deep features of MHSI using CNNs combined with Gabor filters, called the GFCNN model, to enhance the classification of hemocytes under small samples. In subsequent years, Huang et al. [80] further applied MGCNN, an in-depth convolutional network with a Gabor filter classification framework, to classify blood cells. Wei et al. [86] constructed an EtoE-Net model consisting of a two-channel CNN with pixel-by-pixel mapping between the original MHSI as well as the main band images to form globally fused features. Global as well as local features were extracted in the dual-channel CNN, and multiple features were expanded and connected into a superposition vector for fusion. Ultimately, this model has the highest classification performance when compared with traditional machine learning methods. Wang et al. [84] presented a deep-hyper 3D convolutional network that combined 3D-CNN with a 3D attention module in an ultra-deep network for leukocyte classification. The experimental outcomes demonstrated the highest accuracy by placing the attention module at the final layer of the network to classify. Besides the categorization of cells, there are so many medical image classification applications using deep learning in the case of some cancer diagnoses, and most studies have used hyperspectral imaging with convolutional neural network (CNN) classifiers for cancer cell classification [31,33,35,42,85,91,94]. For example, Sommer et al. [34] classified nephrons by using CNNs based on HSI data, specifically by residual neural networks (ResNet). Li et al. [58] used a deep learning architecture with ResNet34 on fluorescent hyperspectral images for the classification of gastric cancers, and the model achieved classification accuracy, specificity, and sensitivity of more than 96%. Bengs et al. [32] investigated in vivo tumor category classification challenges of a more challenging nature using HSI and various deep-learning approaches. A more efficient convolutional gated recurrent unit (CGRU) was used to descend a three-dimensional hyperspectral cube. A CNN following a densely connected convolutional neural network (DenseNet) was then used to handle the two-dimensional data for final classification. Grigoroiu et al. [45] are implementing online classification of data from HSI endoscopy by CNN to stain and analyze different disease stages of the pig esophagus as well as the human esophagus. Spatially distinct colors were shown and validated the properties of deep learning algorithms using color-based classification methods, showing that pixel-level classification is possible for hyperspectral endoscopic data with 18 pure color spectra, reflecting the great potential of CNNs offering color categorization in real-time endoscopic HSI.

The U-net network is the most used and effective network in the medical area. It was initially used only for image segmentation, but later it was gradually used for classification and detection. Since this network is not applicable to the analysis of hyperspectral images, Manifold et al. [95] came up with the U-within-U-Net (UwU-Net) framework, which can classify, segment, and predict orthogonal imaging patterns using various hyperspectral imaging techniques. Prediction of multiple drug locations in rat liver tissue imaging was performed by an external U-Net processing spectral information and internal U-Net processing spatial information.

### 3.3. Detection

Medical hyperspectral images are usually less used in detection and have more potential for development. Usually, in clinical medicine, detection in pathological images is something that can be used in the future as a key part of the diagnosis. One of the papers used a combination of wavelet transform features as well as machine learning, mentioning the use of a discrete wavelet transform (DWT)-based feature classification method [60]. The average spectra of blocks of pixels of the same size were extracted from cancerous as well as normal tissues, respectively, as the original spectra, and a support vector machine (SVM) was used to classify the original data as well as the extracted wavelet features. A tumor mask was generated for the images to distinguish between detecting cancerous as well as normal tissues, and experiments showed better discrimination of overlapping spectra based on wavelet feature classification.

Usually, MHSI applies a CNN network to apply pixel-level classification of medical hyperspectral images to the detection of tumors. There are more detections of head and neck cancers where a two-stream convolutional model [96], with spectral as well as structural branches, was used to detect the hyperspectral data of tongue squamous cell carcinoma obtained from scanning and divided into three regions as tumor, healthy muscle, and epithelium, and finally, the results of the two-stream model outperformed the pure spectral and pure structural methods. Beng et al. [97] advanced a technology to detect in vivo pharyngeal cancer by inputting spectral dimension stacking into a DenseNet2D-MS network used by DenseNet-Blocks with 3D convolutional blocks connected to extract spatial–spectral information. Finally, we detected tumors and healthy tissues after classifying the results using global average pooling (GAP) and classification layers. On the other hand, Halicek et al. [48] used an inception-v4 CNN architecture and introduced a gradient-like activation mapping algorithm to investigate the detection capability with hyperspectral images for cancer detection. It was shown that HSI could help surgeons and pathologists detect tumors in glands. In another paper, a single-stream U-net architecture composed of stacked visible (VIS) and near-infrared (NIR) light was applied [54] to achieve real-time segmentation of hyperspectral imaging in surgery. For the first time, deep-learning semantic segmentation of HSI data was used for tumor detection, and experiments demonstrated the importance of NIR spectroscopy for tumor capture.

In addition to this, it was also used in the detection of other diseases. Examples include breast cancer cell detection through the development of a hyperspectral imaging microscope and deep learning software for digital pathology applications [42]. A manual local feature detection method and the feature detection method grounded in deep learning were adopted for detecting features below the skin surface, demonstrating the ability of the system to track skin features and that the deep-learning skin features were detected and localized better than the local manual features [46].

### 3.4. Segmentation

During medical image segmentation, mostly outlines are sketched out in the image so that the outline of some organs or important parts can be clearly seen as a reference. This operation is important to distinguish some organs in the human body, such as the brain, etc., for medical diagnosis. In recent years, there has been less segmentation for medical hyperspectral images, but the article method is relatively new.

An investigation used a hybrid machine learning, and HSI approach [57] applied to tissue segmentation for image-guided surgery of the liver as well as the thyroid, and seven machine learning models were performed. For each model except U-Net, spatial analysis was performed at three levels: no-spatial analysis; single-scale analysis; and multi-scale analysis. The experimental results for the liver showed that U-Net could identify tissues with high accuracy and achieve optimal segmentation performance. SVM with RBF combined with multi-scale spatial analysis obtained suboptimal performance. In the tissue recognition of HSI data of the thyroid, LR combined with multi-scale spatial analysis segmented with the highest efficiency. Garifullin et al. used dense full convolutional networks (Dense-FCNs) combined with the SegNet model [98] to jointly segment retinal vessels, optic discs, and macula using hyper-spectral retinal images and also experimented on RGB images. The comparison showed that the spectra can provide some additional information about the visual disc and macula and improve recognition performance.

The U-Net architecture is mostly used in medical segmentation, and the main novelty of this architecture is the combination of equal up-sampling layers as well as down-sampling layers, on which most segmentation networks are nowadays improved. Trajanovski et al. [99] segmented squamous cell carcinoma tumors in a U-Net network by randomly selecting 100 patches of 256 × 256 size from each patient’s dataset to be fed into the U-Net network. Due to the selection of larger patch blocks, the spatial background occupies a larger area and provides better performance than pixel-level spectral and structural methods while demonstrating the importance of infrared spectroscopy for the analysis. After that, a single-stream U-Net composed of stacked visible light along with infrared light was published again, confirming the importance of infrared spectroscopy [54]. To make full use of spectral features in 3D hyperspectral data, Wang et al. [55] proposed Hyper-Net, a 3D full convolutional encoding and decoding network for the segmentation of hyperspectral pathology images of melanoma. To preserve the fine features lost due to depth, a dual path was used in the final encoding part with the addition of extended convolutional fast extraction of low-resolution fine-grained features, which significantly improved the segmentation accuracy. Seidlitz et al. [56] combined the visceral tissue oxygen saturation (StO2), near-infrared perfusion index (NPI), tissue water index (TWI), and tissue hemoglobin index (THI) of organic correlation images were overlaid on the cube input model. The neural networks were trained in each of the three input networks according to the studied data granularity levels (pixel-based, super pixel-based, patch-based, and complete image-based). The study demonstrated that the unprocessed HSI data have great advantages in organ segmentation.

In subsequent studies, there is the embedding of a transformer into the coding part of U-Net [100] and applying it in the segmentation of images, which can learn the dense correlation between bands. Having the benefits of both transformers and U-Net, it is more capable of segmenting medical images. However, the acquired information is susceptible to the influence of uncorrelated bands. Therefore, a sparse scheme is introduced to form the spectral transformer SpecTr, which is experimentally shown to be superior to 3D-UNet and 2D-UNet.

**Table 4:** Summary of common deep learning architectures and methods.

References	Architecture									Methods			Detailed method	Applications
	ML	CNN	3D CNN	2D CNN	DenseNet	ResNet	UNet	AlexNet	FCN	Classification	Detection	Segmentation		
[31]			√	√						√			2DCNN + 3DCNN + Inception CNN	Head and neck cancer
[101]		√								√			CNN extracts topological embeddings, and in using binary classification	
[32]					√					√			DenseNet classification after dimensionality reduction using convolutional gated cyclic units	In vivo Tumors
[33]			√	√						√			3D CNN and 2D inception CNN	Head and neck cancer
[63]		√								√			CNN classifier	Head and neck cancer
[34]						√				√			KidneyResNet consisting of Resnet-18	Ambient infusion
[80]		√								√			Combining modulated Gabor and CNN in the MGCNN framework	Red blood cells
[35]		√	√							√			Spectral-Spatial-CNN with 3D convolution	Stomach Cancer
[17]		√								√			CNN training with different patch sizes after PCA dimensionality reduction	Red blood cells
[36]		√								√			Gabor filter and CNN	Red blood cells
[37]		√								√			CNN	Tissue classification
[81]	√		√							√			Compare the classification performance using (RBF-SVM), MLP, and 3DCNN	Stomach and Colon Cancer
[82]	√									√			Combining PCA, SVM, KNN classification with K-means for final weighted voting classification	Brain tumor
[83]	√									√			SVM combined with ANN for classification	Identification of cancer cells
[39]			√	√						√			HybridSpectraNet (HybridSN) composed of 3D CNN and 2D CNN in spectral space	Colon Cancer
[84]			√							√			3D CNN combined with 3D attention module for deep hypernetworks	White blood cells
[40]		√								√			SICSURFIS HSI-CNN system composed of SICSURFIS imager and CNN	Skin disease
[85]	√									√			Stacked auto encoder (SAE)	Tongue coating
[93]		√								√				White blood cells
[41]	√									√			K-means and SAM	Skin disease
[86]		√								√			Two-channel deep fusion network EtoE-Fusion CNN for feature extraction	White and red blood cells
[42]				√						√			Mapping RGB to high broad-spectrum domain with 2D CNN classification	Breast cancer
[95]							√			√			The external U-Net handles spectral information, and the internal u handles spatial information, making up the UwU-Net classification	Drug position
[18]		√								√			Regression-based partitioned deep convolutional networks	Head and neck cancer
[94]	√		√	√						√			1D, 2D, 3D CNN, RNN, MLP, SVM for comparison	Blood Classification
[87]				√	√		√			√			U-Net, 2D CNN, 1D DNN combined with classification	Brain cancer
[43]		√								√			Extracting image elements into patches into CNN	Head and neck cancer
[44]	√									√			RF, SVM, MLP and K-Nearest Neighbor Comparison	Esophageal Cancer
[45]		√								√			Pixel-level classification	Head and neck cancer
[89]	√							√		√			AlexNet combined with SVM	Corneal epithelial tissue
[90]			√	√						√			Hybrid 3D-2D network for extracting spatial and spectral features	Brain cancer
[91]		√								√			CNN with support vector machine (SVM), random forest (RF) synthetic classification	Tissue classification
[102]	√									√			LDA	Septicemia
[48]		√								√			CNN architecture for inception-v4	Head and neck cancer
[103]		√								√			CNN architecture for inception-v4	Head and neck cancer
[51]				√						√			2D CNN classification	Brain cancer
[52]	√									√			RF, logistic regression, SVM comparative classification	Head and neck cancer
[58]						√				√			ResNet34	Stomach Cancer
[92]	√									√			RF, SVM, MLP	Colon Cancer
[59]	√									√			PCA downscaling, Spectral Angle Mapper (SAM)	Stomach Cancer
[60]	√									√			Discrete Wavelet Transform (DWT) based feature extraction, SVM	Head and neck cancer
[96]		√									√		Dual-stream convolution model	Tongue Tumor
[97]			√								√		DenseNet-Blocks combined with 3D CNN to extract spatial spectral information	Head and neck cancer
[46]		√									√		CNN with Deep Local Features (DELF)	Skin Features
[49]		√									√		CNN and SVM + PCA + KNN are used, respectively	Head and neck cancer
[99]							√					√	Select the channel and use U-Net	Head and neck cancer
[55]							√					√	3D full convolutional network with extended convolutional fast and fine-grained feature dual path	Melanoma
[100]							√					√	The encoding part of U-Net uses transformer to extract the spectral information and convolution to extract the spatial information jointly	Carcinoma of bile duct
[56]		√										√	Pixel-based, superpixel-based, patch-based, and full image-based data are fed into the CNN and U-Net, respectively	
[57]							√					√	Seven machine learning models and U-Net were used for the study, respectively	Image-guided surgery
[98]									√			√	SegNet and dense full convolutional neural networks are used	Eye diseases

### 3.5. Conclusions

Through the process of reading and organizing the literature, we conclude that there are some common machine learning and deep learning models in the medical hyperspectral field that can be used many times and show good results. In the models of classification and detection, most of them are improved by using common CNN and Resnet, especially 2D CNN, 3D CNN, and 2D CNN combined with 3D CNN, to extract spatial and spectral features in hyperspectral images. In image segmentation tasks, mostly the classical U-Net’s full convolutional network, are used in a variant to obtain more efficient models.

In the literature, the network models use the Inception multiscale processing module, the 3D attention module, the transformer, the Gabor filter, the discrete wavelet transform (DWT), and the dilated convolution block (DCC) to enhance the feature extraction. These are also some good research directions that can be of great help in subsequently improving the model’s performance.

When calculating the error between the predicted and true values of a model, the loss functions of cross-entropy loss, SoftMax loss, R-square, root mean squared error (RMSE), and mean squared error (MSE) are usually used to measure the degree to which the model fits the data.

## 4. Medical Hyperspectral Image Application Area

### 4.1. Medical Diagnosis

As the resolution of hyperspectral medical images has increased, most of the mainstream research methods now use a combination of spectral features of hyperspectral images and spatial features, which not only extracts the rich spectral information of hyperspectral images but also integrates the extraction of texture structure and detailed information of the images, which greatly improves the classification accuracy. Optical imaging for cancer detection is presented since lesions lead to changes in cell morphology and cause changes in absorption, scattering, and fluorescence properties. So, optical tissue characterization can conversely supply worthy diagnostic messages. HSI can obtain broad-area images of tissues, improving diagnostic accuracy when diagnosing conditions such as stomach, breast, cervical, skin-like diseases, and head and neck. In Table 5, different methods for medical hyperspectral image application areas and a comparison of the different achievements are presented.

#### 4.1.1. Stomach Cancer

Most studies in the last decade or so can play a key part in early cancer detection, and tumor detection can help doctors diagnose cancer and dissect malignant tumor areas when they can be at a safe margin.

Liu et al. [59] used a NIR-HSI system to capture hyperspectral images of gastric tissue and extracted the average spectrum and normal deviation of normally pixeled and post-cancerous image pixels. The dimensionality of the hyper-cube values was squeezed using principal component analysis (PCA), and six were selected as the optimal wavelengths. In addition, the normal and cancerous tissue were categorized using a spectral angle mapper (SAM), and eventually the SAM achieved a classification index of 90% accuracy.

Collins et al. [81] performed detection experiments using the support vector machine with radial basis function kernel (RBF), MLP, and 3DCNN approaches on data containing 12 colon cancer patients as well as 10 esophageal cancer patients, respectively. The final experimental results show that 3DCNN performs better on both datasets. It is also proposed that the use of interactive decision thresholding can be applied in future surgical procedures and be used with high value to improve the classification performance.

Hu et al. [35] built a classification model with an efficient joint CNN to extract tumor deep-spectrum spatial features that facilitate classification. Based upon those differences between gastric cancer organization and regular tissue microscopic hyperspectral features, experiments were conducted on a 30-patient dataset of stomach cancer hyperspectral data. It was demonstrated that the simulation model’s classification rate of both cancerous and natural tissues was more than 97% in accuracy, as well as sensitivity and specificity of gastric cancer tissues.

Li et al. [58] used a fluorescence hyperspectral imaging technique that can obtain spatial as well as spectral information about tissues. They also used a deep learning architecture combined with a spatial–spectral classification method to classify the obtained fluorescence hyperspectral images into non-cancerous lesions, precancerous lesions, and gastric cancer groups, and the accuracy, specificity, and sensitivity of the classification were all above 96%.

#### 4.1.2. Brain Cancer

The most important aspect of cancer surgery in the brain is the accurate excision of the tumor part, which preserves the maximum amount of healthy tissue to ensure the postoperative safety of the patient. Fabelo et al. [87] employed a deep learning-related approach to process highly spectral images of living brain tissue to determine where the tumor is located, which can guide the surgeon in operation. Furthermore, the proposed visualization system can be adjusted at any time and can find the best classification threshold suitable for surgery. Manni et al. [90] they investigated techniques to identify tissue types during surgery and proposed a hybrid 3D–2D CNN architecture based on deep learning-extracted spatial to spectral features to classify normal brain tissue in a live HS image dataset along with glioblastoma tissue. In experiments, it has been shown that the 2D–3D hybrid network has greater precision in the detection of both tumors, vasculature, and healthy ones.

Ortega et al. [51] processed sections of human brain tissue by hematoxylin and eosin (H&E) staining and automatically distinguished glioblastoma (GB) from non-tumor tissue on the sections using HSI and a convolutional neural network (2D-CNN). Experiments were also performed on 13 patients, and the test shows that the mean sensitivity and eigenvalues of the automatic detection of pathological sections using convolutional neural networks on HSI images were higher than those of RGB, indicating the potential of HSI for histopathological analysis.

#### 4.1.3. Head and Neck Cancer

Premature detection of brain tumors in the head and neck is critical to patient survival. Endoscopy is usually used to diagnose disease in the larynx. However, because of the differences in spectral characteristics before and after cancerous lesions, non-invasive detection was performed employing a hyper-spectral imager. 

Maktabi et al. [44] assessed four supervised classifying of algorithms in their experiments: random forest; SVM; MLP; and K-nearest neighbor. HSI recordings of esophageal gastrectomy procedures in 11 patients distinguished between malignant tumors and healthy tissue. The ultimate goal is to obtain real-time tissue recognition techniques in esophagectomy and gastric pull-up procedures.

Zhou et al. [52] developed a novel polarization hyperspectral imaging technique. Normal regions and cancerous regions were distinguished on the Suomy red (H&E) stained head and neck cancer tissue sections. A machine learning framework was used for image classification as well. The outcomes reveal that the SVM classifier has shown the greatest classification precision for both the raw polarized hyperspectral data and the synthetic RGB image data.

Jeyaraj et al. [18] employed a partitioning deep-learning network based on regression for the diagnosis of oral cancer. Two chunking layers were used to label and classify regions of interest in hyperspectral images, and the final classification results were of higher quality than conventional diagnostics.

Halicek et al. [63] used a CNN categorizer for the classification of HSI on resected squamous cell cancer, goiter, and healthy tissue samples of the head and neck. It was also validated by hand annotation by a pathologist specializing in head and neck cancer. Initial results on 50 sufferers show the promise of HSI with DL for automated histological tagging of surgical markers in head and neck patients. Halicek et al. [103] used a DL approach rather than ROI to categorize an entire tissue specimen, using a convolutional network (CNN) to rapidly classify tissue at the carcinoma margins and normal tissue. Further, the potential of HSI-based label-free imaging methods for squamous cell carcinoma detection was investigated for surgical SCC detection. Both CNN and SVM + PCA + KNN were used to generate SCC prediction probability maps [49], respectively, to investigate the information provided by hyperspectral imaging and ML and CNN in head and neck cancer detection and to investigate the limitations of HSI-based and SCC detection. 

#### 4.1.4. Skin Cancer

Leon et al. [41] combined supervised and unsupervised methods to automatically segment the HS map into normal tissue together with pigmented skin lesions (PSL) by a K-means algorithm, and subsequently fed the segmented PSL pixels into a classification framework to classify them as benign as well as malignant tumors. This initial research illustrates in this preliminary study the possibility of the HSI technique to help distinguish benign and malignant PSL for dermatologists in routine clinical practices utilizing live non-invasive handheld devices.

Lindholm et al. [40] utilized a novel hand-held SICSURFIS spectral imager in a study to offer detailed spectral–spatial data. A novel SICSURFIS HSI-CNN system was proposed to effectively distinguish between abnormal and benign skin pathology (melanoma, pigmented nevi, dermatomal nevi, basal cell carcinomas, and squamous cell carcinomas), with good results even for complex skin surfaces.

#### 4.1.5. Eye Diseases

Hadoux et al. [104] identified a noninvasive method for retinal imaging. Due to the significant innate ocular reflectance across individuals and within individuals, between retinal locations, pristine retinal reflectance spectra are useless for differentiating between cases and controls. So, the major axis for spectral variances within groups was removed, and the greatest discrepancy that could be observed among reflex spectra for cases and controls was observed at shorter wavelengths. This method plays an important role in screening for Alzheimer’s disease.

#### 4.1.6. Colon Cancer

Colon cancer is the second most prevalent cancer globally, in addition to being the second cause of cancer-related mortality. Some localized, primary as well as early-stage colon cancers are mainly treated by complete removal of the tumor.

Jansen-Winkein et al. [92] used various machine learning methods in parallel with statistical analysis to assess the potency of HSI to distinguish the mucosa of a healthy colon adenoma from colorectal cancer. The experiments used the hyperbolic tangent function as the activation layer in a neural network to test the supervised classification framework RF/SVM with multilayer perception (MLP). Spatially informative classification was achieved on HSI data using a Gaussian filter with 96% accuracy in classifying mucosal cancer tissue.

Manni et al. [39] used the already proposed 3D-CNN in spectral space as well as the Hybrid Spectra Net (HybridSN) structure of 2D-CNN for classification in six isolated specimens for detection. It ended up with a slightly higher average AUC than the ResNet-based CNN and 3D-CNN. It was also shown that the HybridSN-CNN classification method can be used as an innovative technique for detecting colon cancer tissues and for image-guided colon cancer surgery.

**Table 5:** Comparison of different methods in the field of medical hyperspectral image applications and different achievements.

References	Applications	Different Methods		Different Achievements	
		Machine Learning	Deep learning	Accuracy	Sensitivity
[59]	Stomach cancer	SAM		90%	
[81]			3DCNN	93%	
[35]			CNN	97.57%	97.19%
[58]			ResNet	96.5%	96.6%
[87]	Brain cancer		U-Net, 2D CNN, 1D DNN	94%	
[90]			3D + 2D CNN	80%	
[51]			2D CNN	88%	77%
[44]	Head and neck cancer	Random forest, SVM, MLP, and K-nearest neighbor		63% (SVM)	69% (SVM)
[52]		SVM		93.5%	
[18]			Regression-deep CNN	94.5%	94%
[63]			CNN	96.4%	96.8%
[41]	Skin cancer	K-means, SAM			87.5%
[40]			CNN		93%
[104]	Eye diseases				
[92]	Colon cancer	MLP			86%
[39]			3D + 2D CNN		88%

### 4.2. Conclusion

HSI is still a developing medical imaging modality that can provide spatial and spectral information about some tissue samples. It reflects the quality features such as the size and shape of these samples, as well as their internal texture structure and composition differences, and these rich features provide room for the development of deep learning in medical hyperspectral imaging. Its non-invasive nature also plays a huge role in surgical guidance.

However, because of the fact that the advancement of deep learning is still at the stage of theoretical development and technical exploration in HSI image processing, its application in deep-learning hyperspectral medical diagnosis is limited by the bottleneck of HSI image processing. How to extract richer information at high spectral resolution and spatial resolution without losing some detailed information. It represents a challenge to be tackled in spectral image processing. It is also important to be able to acquire target information quickly and produce diagnostic results since it takes a lot of time from the preprocessing operation of hyperspectral images to the deep learning architecture and final results. As HSI continues to evolve, more experimental studies refine the algorithm and ensure the accountability of HSI analysis for routine clinical use.

## 5. Discussion

### 5.1. Hyperspectral Medical Image Processing vs. Hyperspectral Medical Image Diagnosis

The article discusses some commonly used hyperspectral imaging systems, and introduces the four main methods of spectral imaging: whiskbroom; push broom; staring; and snapshot, and now the new handheld hyperspectral imaging systems. Some common image pre-processing methods are summarized, and the uses of deep learning to classify, detect, and segment hyperspectral images are discussed. Finally, a brief summary of hyperspectral applications in the medical field is given.

Most researchers seek to achieve the best performance of deep learning methods and neural network architectures in a given domain. However, looking at the majority of medical image competitions, it is apparent that relying only on accurate model structures to obtain good analysis results is one-sided. In addition, different data pre-processing methods and data enhancement techniques are also necessary to obtain good scores. Therefore, the pre-processing of hyperspectral images is the most significant step in conducting the research and analysis of hyperspectral images. Since hyperspectral images are acquired in a high number of bands, the images contain a lot of useful information but also cause the images to contain superfluous information such as background and electrical noise, which makes the analysis of the images difficult. Therefore, most studies perform image preprocessing and spectral preprocessing before using the information from the images.

Although some common methods of the image, as well as spectral preprocessing, are discussed in the paper, some limitations of these methods exist in the application process, and the most suitable methods should be investigated in continuous practice. In the process of data collection, new preprocessing methods for Fourier transform and wavelet transform were found, which were capable of frequency domain and time domain conversion and showed good performance in analysis. These new data pretreatment methods provide a useful direction for future research in data analysis and a good basis for research development in other fields as well.

### 5.2. Challenges and Opportunities

The majority of current research has shown that spectral images can better extract diagnostic data of relevant tissue physiological, morphological, and compositional information. Although there is potential for the early diagnosis of diseases, there are still many limitations to the research that hinder the advancement of deep learning in the medical domain.

Firstly, research on medical images in deep learning has been conducted by fewer teams and in a narrower scope. Most of the research is to classify cells or some tissue samples by CNN to determine whether they have cancer or not. The development of some advanced algorithms can more accurately distinguish the categories of tissues, and the research in this area is still slow to develop in terms of applications.

Secondly, there is an extreme lack of data for the analysis of medical images in deep-learning applications. There are often limitations in the calibrated datasets, resulting in poor training and classification performance of experiments, and some publicly available datasets are scarce and small, and high-quality data calibration is lacking. Although this problem can be solved by data augmentation, there is a risk of overfitting. Nowadays, most of the general computer vision tasks are solved by applying smaller filters at a deeper level or by hyperparameter optimization.

Finally, most of the hyperspectral acquisition devices are now relatively large, with fewer applications for some handheld devices, and their application to deep learning, combined with algorithms for real-time medical analysis, has yet to be developed. With the maturity of the technology, it can realize the convenient situation where the analysis of tissue cases can be more quickly and safely applied in the clinic.

Although there are many problems that hinder the development of this field, as technology continues to develop, more and more teams will devote themselves to research in the field of medical hyper-spectroscopy and build more complete databases to develop more convenient and efficient imaging spectrometers. In addition, more scholars will study the method of combining spectral imaging with other biomedical imaging. This will make the analysis more comprehensive and help to interpret the parameter information of different biological tissues to replace the traditional diagnostic equipment.

Therefore, in the process of summarizing and integrating the articles, we should choose some meaningful articles and methods to describe and synthesize so that they can be more representative and provide some directions for later researchers. However, theoretical research is indispensable, and practical applicability is also important for the evolution of the field. Publicly available datasets facilitate the aggregation of research results. It is not surprising that studies in brain cancer diagnosis and diabetic podiatry have shown that complete, labeled datasets can increase the attention of researchers in this direction. It is expected that easily extractable data labels will become more readily accessible in the future.

In this review, a large volume of literature was collated, and through the examination of different focuses, some existing studies were divided into different categories, and the articles were summarized in different sections to present a clear framework that reflects the development of HSI and the development of the combination of different technologies. This development occurred through the research on the application of HSI in clinical analysis and operation guidance, to the analysis and judgment of medical HSI images combined with machine learning, and finally to the applications of deep learning. An increasing number of scholars have devoted themselves to this research, which has also greatly advanced the development of this field.

### 5.3. Datasets

Most of the authors did not give public experimental data and codes due to privacy or medical ethics’ principles. However, there are still a few institutions that provide relevant datasets. Although there are some hyperspectral image data belonging to animal tissues, it is useful to promote the research of algorithms and models. We have listed the collected public datasets whenever possible.

1. HSI Human Brain Database

Website: https://hsibraindatabase.iuma.ulpgc.es/ (accessed on 19 March 2018)

The links allow the download of the hyperspectral images of in vivo human brain employed in the paper [38]. This dataset has been used in several papers and is currently the most popular hyperspectral public dataset;

2. MALDI rat liver anticancer drug spiked-in dataset (imzML)

Website: https://www.ebi.ac.uk/pride/archive/projects/PXD016146 (accessed on 11 June 2019)

3. The Hyperspectral SRS and Fluorescence data

Website: https://figshare.com/articles/dataset/hs_SRS_fluo_images_zip/13497138 (accessed on 29 December 2020)

The links allow the download of the hyperspectral SRS and corresponding organelle fluorescence images used in training deep-learning prediction models using U-within-U-Net.

Links 2 and 3 are from reference [95]. The authors also shared the source code:

Source code: https://github.com/B-Manifold/pytorch_fnet_UwUnet (accessed on 29 December 2020)

4. A clinically translatable hyperspectral endoscopy (HySE) system for imaging the gastrointestinal tract

Website: https://www.repository.cam.ac.uk/handle/1810/270691 (accessed on 17 January 2018)

The links allow the download of the raw and processed data of simulation and experiments in the paper (a clinically translatable hyperspectral endoscopy (HySE) system for imaging the gastrointestinal tract). This dataset was obtained from reference [61];

5. Parallel Implementations Assessment of a Spatial–Spectral Classifier for Hyperspectral Clinical Applications

Website: https://ieee-dataport.org/open-access/dataset-parallel-implementations-assessment-spatial-spectral-classifier-hyperspectral (accessed on 17 May 2022)

The links allow the download of HS images taken from dermatological interventions. This is a very new dataset provided by Himar Fabelo et al. (the authors of references [38,42,47,49,51]);

6. Microscopic Hyperspectral Choledoch Dataset

Website: https://www.kaggle.com/datasets/ethelzq/multidimensional-choledoch-database (accessed on 12 December 2017)

The links allow the download of a dataset for both microscopy hyperspectral and color images of cholangiocarcinoma. This dataset is presented in reference [100]. Due to the upload space limitation, providers only uploaded part of the data. The original files are located here: http://bio-hsi.ecnu.edu.cn/. This dataset requires a request to obtain all data;

7. Multispectral Imaging Dataset of Colorectal tissue

Website: https://figshare.com/articles/figure/Multispectral_Imaging_Dataset_of_Colorectal_tissue/6224957/1 (accessed on 5 June 2018)

The links allow the download of images of two benign abnormality classes along with normal and cancerous classes. The dataset consists of four classes, each represented by infra-red spectrum bands in addition to the visual spectrum bands. This dataset is presented in reference [51].

## Data Availability

Not applicable.
